# Supplementary material for: Adaptation of metal and antibiotic resistant traits in novel β-Proteobacterium Achromobacter xylosoxidans BHW-15
Source: PeerJ. 2019 Mar 13;7:e6537. doi: 10.7717/peerj.6537 (PMC6421061; doi:10.7717/peerj.6537)
Supplement: Supplemental Information 2 [file peerj-07-6537-s002.doc]

**Secondary Metabolite Analysis of *A. xylosoxidans* BHW-15 by Anti-SMASH**

| **Input accession number** | **Position** | **Gene cluster type** | **Detected Gene cluster genes in the Database** |
| --- | --- | --- | --- |
| c00162_NODE_64.. | NODE_647_length_3209_cov_10.5127_ID_1293 | Ectoine | ctg162_1;ctg162_2;ctg162_3;ctg162_4;ctg162_5 |
| c00420_NODE_17.. | NODE_17_length_15877_cov_6.81023_ID_33 | Ectoine | ctg420_2;ctg420_3;ctg420_4;ctg420_5;ctg420_6;ctg420_7;ctg420_8;ctg420_9;ctg420_10;ctg420_11;ctg420_12;ctg420_13;ctg420_14 |
| c00519_NODE_56.. | NODE_564_length_3672_cov_8.88717_ID_1127 | Resorcinol | ctg519_1;ctg519_2;ctg519_3;ctg519_4 |
| c01114_NODE_11.. | NODE_1101_length_1700_cov_2.3096_ID_2201 | Phosphonate | ctg1114_1;ctg1114_2;ctg1114_3 |
| c00532_NODE_23.. | NODE_232_length_6727_cov_5.9953_ID_463 | arylpolyene | ctg532_1;ctg532_2;ctg532_3;ctg532_4;ctg532_5;ctg532_6;ctg532_7;ctg532_8 |
| c00570_NODE_49.. | NODE_493_length_3997_cov_4.34238_ID_985 | Terpene | ctg570_1;ctg570_2;ctg570_3;ctg570_4 |
| c00234_NODE_56.. | NODE_565_length_3664_cov_5.86037_ID_1129 | cf_putative | ctg234_1;ctg234_2;ctg234_3;ctg234_4;ctg234_5 |
| c00283_NODE_49.. | NODE_497_length_3983_cov_2.90301_ID_993 | cf_putative | ctg283_1;ctg283_2;ctg283_3;ctg283_4;ctg283_5;ctg283_6 |
| c00430_NODE_13.. | NODE_133_length_8708_cov_5.35629_ID_265 | cf_putative | ctg430_4;ctg430_5;ctg430_6;ctg430_7;ctg430_8;ctg430_9;ctg430_10;ctg430_11;ctg430_12;ctg430_13 |
| c00442_NODE_17.. | NODE_174_length_7847_cov_7.39598_ID_347 | cf_putative | ctg442_3;ctg442_4;ctg442_5;ctg442_6;ctg442_7;ctg442_8 |
| c00513_NODE_9_.. | NODE_9_length_18093_cov_8.79516_ID_17 | cf_saccharide | ctg513_1;ctg513_2;ctg513_3;ctg513_4;ctg513_5;ctg513_6;ctg513_7;ctg513_8;ctg513_9;ctg513_10;ctg513_11;ctg513_12;ctg513_13;ctg513_14;ctg513_15 |
| c00526_NODE_10.. | NODE_104_length_10266_cov_7.90394_ID_207 | cf_putative | ctg526_1;ctg526_2;ctg526_3;ctg526_4;ctg526_5 |
| c00579_NODE_22.. | NODE_22_length_15179_cov_27.7443_ID_43 | cf_saccharide | ctg579_1;ctg579_2;ctg579_3;ctg579_4;ctg579_5;ctg579_6;ctg579_7;ctg579_8;ctg579_9;ctg579_10;ctg579_11;ctg579_12;ctg579_13;ctg579_14 |
| c00602_NODE_23.. | NODE_23_length_15143_cov_8.42399_ID_45 | cf_putative | ctg602_2;ctg602_3;ctg602_4;ctg602_5;ctg602_6;ctg602_7;ctg602_8;ctg602_9 |
| c00721_NODE_15.. | NODE_151_length_8317_cov_9.09707_ID_301 | cf_putative | ctg721_4;ctg721_5;ctg721_6;ctg721_7;ctg721_8 |
| c00934_NODE_56.. | NODE_569_length_3631_cov_10.7657_ID_1137 | cf_fatty_acid | ctg934_1;ctg934_2;ctg934_3;ctg934_4;ctg934_5 |
| c00957_NODE_35.. | NODE_35_length_14585_cov_7.97635_ID_69 | cf_saccharide | ctg957_1;ctg957_2;ctg957_3;ctg957_4;ctg957_5;ctg957_6;ctg957_7;ctg957_8;ctg957_9;ctg957_10 |
| c01376_NODE_25.. | NODE_258_length_6275_cov_4.69099_ID_515 | cf_saccharide | ctg1376_1;ctg1376_2;ctg1376_3;ctg1376_4;ctg1376_5;ctg1376_6;ctg1376_7;ctg1376_8;ctg1376_9;ctg1376_10 |
| c01394_NODE_12.. | NODE_1284_length_1299_cov_4.6058_ID_2567 | cf_fatty_acid | ctg1394_1;ctg1394_2 |
